# Chloridobis[*N*′-(2-meth­oxy­benzyl­idene)-4-nitro­benzohydrazidato-κ^2^ *O*,*N*′](4-methyl­pyridine-κ*N*)cobalt(III)

**DOI:** 10.1107/S1600536811003321

**Published:** 2011-02-02

**Authors:** Qiong-Jie Wu, Xiao-Hua Chen, Jiang Jiang, Bi-Qiong Cai, Yong-Ping Xie

**Affiliations:** a College of Life Science, Fujian Agriculture and Forestry University, Fuzhou, Fujian 350002, People’s Republic of China; b College of Chemistry and Materials Science, Fujian Normal University, Fuzhou, Fujian 350007, People’s Republic of China

## Abstract

In the title complex, [Co(C_15_H_12_N_3_O_4_)_2_Cl(C_6_H_7_N)], the Co^III^ ion is coordinated by two N atoms and two O atoms from two deprotonated Schiff base ligands, one N atom from a 4-methyl­pyridine ligand and one Cl atom, forming a distorted octa­hedral geometry. The Co^III^ ion is displaced by 0.038 (2) Å from the equatorial plane towards the axial Cl atom.

## Related literature

For general background to aroylhydrazines and their metal complexes, see: Cariati *et al.* (2002 ▶); Chen *et al.* (2010 ▶); Fun *et al.* (1996 ▶); Liao *et al.* (2000 ▶); Liu & Gao (1998 ▶); Lu *et al.* (1996 ▶); Tai *et al.* (2003 ▶); Xue & Liu (2006 ▶); Yang & Pan (2004 ▶). For related structures, see: Chen & Liu (2006 ▶); Tan *et al.* (2010 ▶); Wu & Liu (2004 ▶).
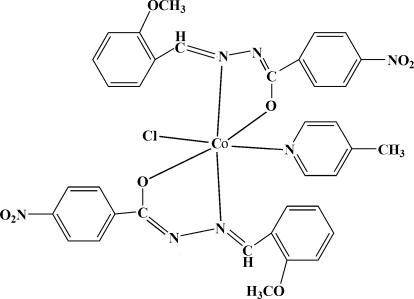


## Experimental

### 

#### Crystal data

[Co(C_15_H_12_N_3_O_4_)_2_Cl(C_6_H_7_N)]
*M* *_r_* = 784.06
Triclinic, 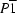

*a* = 10.530 (2) Å
*b* = 14.028 (3) Å
*c* = 14.794 (3) Å
α = 62.203 (3)°
β = 85.669 (3)°
γ = 72.275 (3)°
*V* = 1835.6 (7) Å^3^
*Z* = 2
Mo *K*α radiation
μ = 0.60 mm^−1^
*T* = 293 K
0.12 × 0.10 × 0.08 mm

#### Data collection

Rigaku R-AXIS RAPID diffractometer
Absorption correction: multi-scan (*ABSCOR*; Higashi, 1995 ▶) *T*_min_ = 0.931, *T*_max_ = 0.954
12688 measured reflections
6308 independent reflections
3144 reflections with *I* > 2σ(*I*)
*R*_int_ = 0.064

#### Refinement

*R*[*F* ^2^ > 2σ(*F* ^2^)] = 0.063
*wR*(*F* ^2^) = 0.157
*S* = 0.94
6308 reflections
478 parameters
H-atom parameters constrained
Δρ_max_ = 0.73 e Å^−3^
Δρ_min_ = −0.29 e Å^−3^

### 

Data collection: *RAPID-AUTO* (Rigaku, 1998 ▶); cell refinement: *RAPID-AUTO*; data reduction: *CrystalStructure* (Rigaku/MSC, 2002 ▶); program(s) used to solve structure: *SHELXS97* (Sheldrick, 2008 ▶); program(s) used to refine structure: *SHELXL97* (Sheldrick, 2008 ▶); molecular graphics: *DIAMOND* (Brandenburg, 1999 ▶); software used to prepare material for publication: *SHELXTL* (Sheldrick, 2008 ▶).

## Supplementary Material

Crystal structure: contains datablocks I, global. DOI: 10.1107/S1600536811003321/hy2400sup1.cif

Structure factors: contains datablocks I. DOI: 10.1107/S1600536811003321/hy2400Isup2.hkl

Additional supplementary materials:  crystallographic information; 3D view; checkCIF report

## Figures and Tables

**Table 1:** Selected bond lengths (Å)

Co1—O3	1.887 (3)
Co1—O7	1.882 (3)
Co1—N3	1.917 (4)
Co1—N6	1.932 (4)
Co1—N7	1.983 (4)
Co1—Cl1	2.2472 (17)

**Fig. 1.**
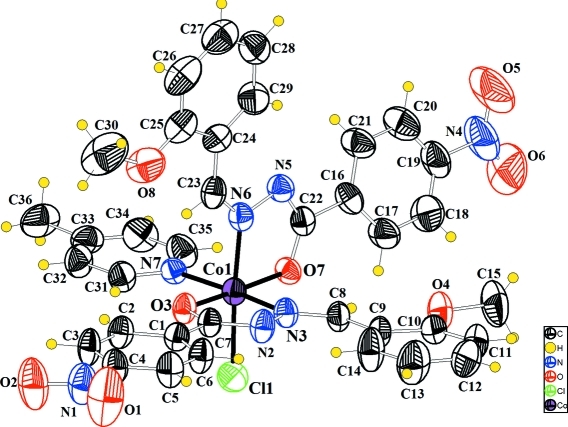
The molecular structure of the title compound, showing 50% probability displacement ellipsoids.

## supplementary crystallographic information

### Comment

Recently, much attention has been paid to the chemistry of aroylhydrazines and their complexes with metal ions (Cariati *et al.*, 2002; Chen *et al.*, 2010; Liu & Gao 1998; Tai *et al.*, 2003; Xue & Liu, 2006). These compounds can serve as potential chelating agents (Fun *et al.*, 1996; Lu *et al.*, 1996) and possess biological activity (Liao *et al.*, 2000; Yang & Pan, 2004). Here we report the synthesis and crystal structure of the title compound.

As shown in Fig. 1, the Co^III^ ion exists in a distorted octahedral N_3_O_2_Cl coordination geometry. The equatorial plane is defined by three donor atoms (O3, O7 and N3) from two hydrazine ligands and N7 atom from a 4-methylpyridine ligand, with an r.m.s. deviation of 0.0305 Å from the mean plane. The axial sites are occupied by N6 of one hydrazine ligand and Cl1. The Co^III^ ion is displaced towards the axial Cl1 atom by 0.038 (2)Å from the equatorial plane. Bond distances (Table 1) and bond angles around Co1 atom are compared with those in the reported cobalt complexes (Chen & Liu, 2006; Tan *et al.*, 2010; Wu & Liu, 2004).

### Experimental

The hydrazine ligand (HL) was prepared by the reaction of *o*-methoxybenzaldehyde and *p*-nitrobenzoylhydrazine in a molar ratio of 1:1 under reflux in ethanol for 3 h. The yellow product obtained on cooling was recrystallized from methanol. To HL (1 mmol) in DMF (5 ml) was added an equimolar amount of CoCl_2_ in methanol (5 ml). After stirring for 15 min, 0.2 ml *p*-methylpyridine was added to the solution. The resulting mixture was stirred at room temperature for an additional period of 1 h and then filtered. Brown prism-shaped crystals were obtained from the solution after two weeks. Analysis, calculated for C_36_H_31_ClCoN_7_O_8_: C 54.98, H 4.01, N 12.43%; found: C 55.10, H 3.95, N 12.50%.

### Refinement

H atoms were placed at calculated positions and treated as riding on their parent atoms, with C—H = 0.93 (CH) and 0.96 (CH_3_) Å and with *U*_iso_(H) = 1.2(1.5 for methyl)*U*_eq_(C).

### Crystal data

**Table:**

[Co(C_15_H_12_N_3_O_4_)_2_Cl(C_6_H_7_N)]	*Z* = 2
*M**_r_* = 784.06	*F*(000) = 808
Triclinic, *P*1	*D*_x_ = 1.419 Mg m^−^^3^
Hall symbol: -P 1	Mo *K*α radiation, λ = 0.71073 Å
*a* = 10.530 (2) Å	Cell parameters from 3144 reflections
*b* = 14.028 (3) Å	θ = 2.0–25.0°
*c* = 14.794 (3) Å	µ = 0.60 mm^−^^1^
α = 62.203 (3)°	*T* = 293 K
β = 85.669 (3)°	Prism, brown
γ = 72.275 (3)°	0.12 × 0.10 × 0.08 mm
*V* = 1835.6 (7) Å^3^	

### Data collection

**Table:**

Rigaku R-AXIS RAPID diffractometer	6308 independent reflections
Radiation source: rotation anode	3144 reflections with *I* > 2σ(*I*)
graphite	*R*_int_ = 0.064
ω scans	θ_max_ = 25.0°, θ_min_ = 2.0°
Absorption correction: multi-scan (*ABSCOR*; Higashi, 1995)	*h* = −12→12
*T*_min_ = 0.931, *T*_max_ = 0.954	*k* = −16→14
12688 measured reflections	*l* = −17→17

### Refinement

**Table:**

Refinement on *F*^2^	Primary atom site location: structure-invariant direct methods
Least-squares matrix: full	Secondary atom site location: difference Fourier map
*R*[*F*^2^ > 2σ(*F*^2^)] = 0.063	Hydrogen site location: inferred from neighbouring sites
*wR*(*F*^2^) = 0.157	H-atom parameters constrained
*S* = 0.94	*w* = 1/[σ^2^(*F*_o_^2^) + (0.0697*P*)^2^] where *P* = (*F*_o_^2^ + 2*F*_c_^2^)/3
6308 reflections	(Δ/σ)_max_ = 0.001
478 parameters	Δρ_max_ = 0.73 e Å^−^^3^
0 restraints	Δρ_min_ = −0.29 e Å^−^^3^

### Fractional atomic coordinates and isotropic or equivalent isotropic displacement parameters (Å^2^)

**Table:**

	*x*	*y*	*z*	*U*_iso_*/*U*_eq_	
Co1	0.37392 (7)	0.10485 (6)	0.26462 (5)	0.0404 (2)	
Cl1	0.56482 (14)	−0.04002 (12)	0.31714 (10)	0.0594 (4)	
O1	0.7244 (6)	0.5110 (5)	0.4349 (4)	0.128 (2)	
O2	0.6499 (5)	0.4123 (4)	0.5748 (4)	0.1036 (16)	
O3	0.4228 (3)	0.1646 (3)	0.3428 (2)	0.0454 (9)	
O4	0.4954 (4)	0.2019 (3)	−0.1040 (3)	0.0698 (11)	
O5	−0.0960 (7)	0.1461 (6)	−0.2398 (5)	0.145 (3)	
O6	0.0849 (7)	0.0090 (6)	−0.2185 (5)	0.134 (2)	
O7	0.3203 (3)	0.0612 (3)	0.1748 (2)	0.0427 (8)	
O8	0.1019 (4)	0.4428 (4)	0.3188 (4)	0.0871 (14)	
N1	0.6684 (6)	0.4408 (5)	0.4845 (4)	0.0769 (16)	
N2	0.5113 (4)	0.2641 (4)	0.1940 (3)	0.0493 (11)	
N3	0.4654 (4)	0.2001 (3)	0.1626 (3)	0.0439 (10)	
N4	0.0158 (9)	0.0820 (8)	−0.1975 (5)	0.103 (2)	
N5	0.1387 (4)	0.2219 (4)	0.1352 (3)	0.0468 (11)	
N6	0.2066 (4)	0.2250 (4)	0.2103 (3)	0.0435 (11)	
N7	0.2841 (4)	0.0135 (3)	0.3809 (3)	0.0409 (10)	
C1	0.5279 (5)	0.2942 (4)	0.3384 (4)	0.0439 (13)	
C2	0.4979 (5)	0.2702 (4)	0.4383 (4)	0.0496 (14)	
H2B	0.4478	0.2211	0.4725	0.060*	
C3	0.5420 (5)	0.3184 (5)	0.4871 (4)	0.0558 (15)	
H3A	0.5225	0.3027	0.5540	0.067*	
C4	0.6163 (6)	0.3909 (5)	0.4334 (4)	0.0527 (14)	
C5	0.6444 (6)	0.4179 (5)	0.3343 (4)	0.0570 (15)	
H5B	0.6917	0.4693	0.2996	0.068*	
C6	0.6020 (5)	0.3684 (5)	0.2872 (4)	0.0543 (15)	
H6A	0.6227	0.3843	0.2204	0.065*	
C7	0.4835 (5)	0.2372 (4)	0.2892 (4)	0.0425 (12)	
C8	0.4890 (5)	0.2082 (4)	0.0719 (3)	0.0481 (14)	
H8A	0.4581	0.1616	0.0565	0.058*	
C9	0.5563 (5)	0.2793 (5)	−0.0091 (4)	0.0532 (14)	
C10	0.5598 (6)	0.2723 (5)	−0.1022 (4)	0.0565 (15)	
C11	0.6177 (6)	0.3369 (6)	−0.1841 (4)	0.0749 (19)	
H11A	0.6169	0.3330	−0.2450	0.090*	
C12	0.6774 (7)	0.4083 (7)	−0.1765 (5)	0.090 (2)	
H12A	0.7174	0.4516	−0.2324	0.108*	
C13	0.6782 (7)	0.4160 (6)	−0.0861 (5)	0.091 (2)	
H13A	0.7186	0.4641	−0.0813	0.109*	
C14	0.6187 (7)	0.3515 (6)	−0.0040 (4)	0.0764 (19)	
H14A	0.6200	0.3562	0.0566	0.092*	
C15	0.4867 (8)	0.1978 (6)	−0.1990 (5)	0.100 (2)	
H15A	0.4389	0.1461	−0.1904	0.151*	
H15B	0.5752	0.1726	−0.2179	0.151*	
H15C	0.4403	0.2718	−0.2520	0.151*	
C16	0.1556 (6)	0.1187 (4)	0.0397 (4)	0.0505 (14)	
C17	0.2401 (6)	0.0517 (5)	0.0012 (4)	0.0685 (17)	
H17A	0.3286	0.0142	0.0279	0.082*	
C18	0.1936 (7)	0.0404 (6)	−0.0764 (5)	0.0777 (19)	
H18A	0.2501	−0.0052	−0.1019	0.093*	
C19	0.0650 (8)	0.0960 (6)	−0.1154 (5)	0.0727 (19)	
C20	−0.0214 (7)	0.1636 (6)	−0.0792 (5)	0.0756 (19)	
H20A	−0.1092	0.2016	−0.1075	0.091*	
C21	0.0238 (6)	0.1743 (5)	−0.0009 (4)	0.0667 (17)	
H21A	−0.0339	0.2189	0.0250	0.080*	
C22	0.2089 (6)	0.1335 (5)	0.1225 (4)	0.0441 (13)	
C23	0.1556 (6)	0.3046 (5)	0.2358 (4)	0.0490 (14)	
H23A	0.2047	0.2987	0.2888	0.059*	
C24	0.0358 (5)	0.4014 (5)	0.1967 (4)	0.0472 (13)	
C25	0.0126 (7)	0.4728 (5)	0.2425 (5)	0.0591 (16)	
C26	−0.0976 (8)	0.5700 (6)	0.2070 (6)	0.077 (2)	
H26A	−0.1120	0.6183	0.2359	0.093*	
C27	−0.1858 (7)	0.5946 (6)	0.1285 (6)	0.0777 (19)	
H27A	−0.2587	0.6600	0.1049	0.093*	
C28	−0.1675 (7)	0.5250 (6)	0.0855 (5)	0.0713 (18)	
H28A	−0.2287	0.5417	0.0341	0.086*	
C29	−0.0568 (6)	0.4283 (5)	0.1188 (4)	0.0626 (16)	
H29A	−0.0442	0.3811	0.0888	0.075*	
C30	0.0780 (8)	0.5059 (8)	0.3742 (7)	0.133 (3)	
H30A	0.1499	0.4739	0.4260	0.199*	
H30B	−0.0047	0.5033	0.4059	0.199*	
H30C	0.0728	0.5831	0.3280	0.199*	
C31	0.2877 (5)	0.0132 (5)	0.4712 (4)	0.0539 (15)	
H31A	0.3369	0.0541	0.4792	0.065*	
C32	0.2223 (5)	−0.0445 (5)	0.5528 (4)	0.0597 (16)	
H32A	0.2257	−0.0399	0.6133	0.072*	
C33	0.1520 (5)	−0.1088 (5)	0.5453 (5)	0.0617 (16)	
C34	0.1506 (6)	−0.1112 (5)	0.4531 (5)	0.0687 (18)	
H34A	0.1052	−0.1541	0.4445	0.082*	
C35	0.2171 (6)	−0.0495 (5)	0.3735 (4)	0.0632 (16)	
H35A	0.2149	−0.0522	0.3121	0.076*	
C36	0.0757 (6)	−0.1717 (5)	0.6334 (5)	0.088 (2)	
H36A	0.0882	−0.1598	0.6905	0.131*	
H36B	0.1087	−0.2512	0.6534	0.131*	
H36C	−0.0178	−0.1439	0.6116	0.131*	

### Atomic displacement parameters (Å^2^)

**Table:**

	*U*^11^	*U*^22^	*U*^33^	*U*^12^	*U*^13^	*U*^23^
Co1	0.0446 (5)	0.0485 (5)	0.0383 (4)	−0.0235 (4)	0.0059 (3)	−0.0229 (3)
Cl1	0.0574 (9)	0.0657 (10)	0.0590 (9)	−0.0195 (8)	0.0010 (7)	−0.0313 (8)
O1	0.187 (6)	0.188 (6)	0.110 (4)	−0.154 (5)	0.063 (4)	−0.097 (4)
O2	0.162 (5)	0.130 (4)	0.067 (3)	−0.087 (4)	0.019 (3)	−0.060 (3)
O3	0.053 (2)	0.058 (2)	0.0385 (19)	−0.032 (2)	0.0124 (16)	−0.0246 (17)
O4	0.097 (3)	0.080 (3)	0.047 (2)	−0.029 (3)	0.009 (2)	−0.041 (2)
O5	0.149 (6)	0.187 (6)	0.124 (5)	−0.029 (5)	−0.057 (4)	−0.095 (5)
O6	0.157 (6)	0.192 (7)	0.131 (5)	−0.073 (5)	0.017 (4)	−0.127 (5)
O7	0.045 (2)	0.047 (2)	0.043 (2)	−0.0155 (19)	−0.0015 (17)	−0.0251 (17)
O8	0.073 (3)	0.118 (4)	0.117 (4)	−0.027 (3)	0.008 (3)	−0.094 (3)
N1	0.102 (4)	0.101 (4)	0.063 (4)	−0.055 (4)	0.018 (3)	−0.054 (3)
N2	0.065 (3)	0.057 (3)	0.044 (3)	−0.035 (3)	0.014 (2)	−0.030 (2)
N3	0.055 (3)	0.047 (3)	0.039 (2)	−0.022 (2)	0.005 (2)	−0.024 (2)
N4	0.125 (7)	0.135 (7)	0.074 (4)	−0.060 (6)	−0.016 (4)	−0.053 (5)
N5	0.048 (3)	0.050 (3)	0.047 (3)	−0.015 (2)	−0.001 (2)	−0.026 (2)
N6	0.049 (3)	0.048 (3)	0.037 (2)	−0.021 (2)	0.005 (2)	−0.019 (2)
N7	0.040 (3)	0.045 (3)	0.040 (2)	−0.018 (2)	0.0019 (19)	−0.018 (2)
C1	0.048 (3)	0.051 (3)	0.042 (3)	−0.024 (3)	0.007 (2)	−0.025 (3)
C2	0.063 (4)	0.050 (3)	0.045 (3)	−0.031 (3)	0.016 (3)	−0.023 (3)
C3	0.079 (4)	0.072 (4)	0.042 (3)	−0.040 (3)	0.018 (3)	−0.038 (3)
C4	0.072 (4)	0.060 (4)	0.045 (3)	−0.035 (3)	0.007 (3)	−0.031 (3)
C5	0.075 (4)	0.067 (4)	0.048 (3)	−0.043 (3)	0.016 (3)	−0.030 (3)
C6	0.070 (4)	0.068 (4)	0.046 (3)	−0.041 (3)	0.018 (3)	−0.034 (3)
C7	0.050 (3)	0.045 (3)	0.035 (3)	−0.022 (3)	0.009 (2)	−0.018 (2)
C8	0.055 (3)	0.061 (4)	0.036 (3)	−0.024 (3)	0.009 (3)	−0.026 (3)
C9	0.062 (4)	0.061 (4)	0.038 (3)	−0.020 (3)	0.009 (3)	−0.024 (3)
C10	0.057 (4)	0.060 (4)	0.041 (3)	−0.008 (3)	0.003 (3)	−0.020 (3)
C11	0.075 (5)	0.103 (5)	0.041 (4)	−0.026 (4)	0.016 (3)	−0.030 (4)
C12	0.078 (5)	0.113 (6)	0.055 (4)	−0.041 (5)	0.027 (4)	−0.017 (4)
C13	0.111 (6)	0.111 (6)	0.073 (5)	−0.074 (5)	0.036 (4)	−0.041 (4)
C14	0.111 (5)	0.099 (5)	0.053 (4)	−0.076 (5)	0.030 (3)	−0.038 (4)
C15	0.139 (7)	0.122 (6)	0.057 (4)	−0.037 (5)	0.004 (4)	−0.057 (4)
C16	0.065 (4)	0.047 (3)	0.046 (3)	−0.024 (3)	−0.006 (3)	−0.021 (3)
C17	0.064 (4)	0.084 (5)	0.072 (4)	−0.010 (4)	−0.011 (3)	−0.053 (4)
C18	0.082 (5)	0.094 (5)	0.082 (5)	−0.027 (4)	0.004 (4)	−0.062 (4)
C19	0.084 (5)	0.098 (5)	0.056 (4)	−0.044 (5)	−0.004 (4)	−0.040 (4)
C20	0.073 (5)	0.086 (5)	0.083 (5)	−0.028 (4)	−0.017 (4)	−0.046 (4)
C21	0.060 (4)	0.076 (5)	0.073 (4)	−0.021 (4)	−0.011 (3)	−0.040 (4)
C22	0.049 (4)	0.052 (4)	0.038 (3)	−0.028 (3)	0.007 (3)	−0.020 (3)
C23	0.063 (4)	0.052 (4)	0.045 (3)	−0.027 (3)	0.013 (3)	−0.029 (3)
C24	0.047 (4)	0.043 (3)	0.054 (3)	−0.020 (3)	0.011 (3)	−0.023 (3)
C25	0.064 (4)	0.058 (4)	0.074 (4)	−0.033 (4)	0.023 (4)	−0.039 (4)
C26	0.085 (5)	0.062 (5)	0.103 (6)	−0.034 (4)	0.040 (5)	−0.051 (4)
C27	0.075 (5)	0.059 (5)	0.084 (5)	−0.015 (4)	0.021 (4)	−0.027 (4)
C28	0.075 (5)	0.066 (5)	0.061 (4)	−0.011 (4)	0.000 (3)	−0.026 (4)
C29	0.073 (4)	0.051 (4)	0.060 (4)	−0.012 (4)	0.003 (3)	−0.025 (3)
C30	0.099 (6)	0.204 (9)	0.186 (9)	−0.052 (6)	0.032 (6)	−0.163 (8)
C31	0.054 (4)	0.067 (4)	0.046 (3)	−0.032 (3)	0.009 (3)	−0.023 (3)
C32	0.068 (4)	0.063 (4)	0.046 (3)	−0.032 (4)	0.014 (3)	−0.019 (3)
C33	0.048 (4)	0.057 (4)	0.060 (4)	−0.016 (3)	0.007 (3)	−0.012 (3)
C34	0.065 (4)	0.069 (4)	0.072 (4)	−0.042 (4)	0.007 (3)	−0.020 (4)
C35	0.069 (4)	0.071 (4)	0.056 (4)	−0.034 (4)	−0.002 (3)	−0.026 (3)
C36	0.064 (4)	0.073 (5)	0.081 (4)	−0.026 (4)	0.024 (3)	−0.001 (4)

### Geometric parameters (Å, °)

**Table:**

Co1—O3	1.887 (3)	C12—H12A	0.9300
Co1—O7	1.882 (3)	C13—C14	1.375 (7)
Co1—N3	1.917 (4)	C13—H13A	0.9300
Co1—N6	1.932 (4)	C14—H14A	0.9300
Co1—N7	1.983 (4)	C15—H15A	0.9600
Co1—Cl1	2.2472 (17)	C15—H15B	0.9600
O1—N1	1.213 (6)	C15—H15C	0.9600
O2—N1	1.221 (6)	C16—C17	1.384 (7)
O3—C7	1.281 (5)	C16—C21	1.387 (7)
O4—C10	1.367 (7)	C16—C22	1.507 (7)
O4—C15	1.445 (6)	C17—C18	1.371 (7)
O5—N4	1.235 (8)	C17—H17A	0.9300
O6—N4	1.218 (8)	C18—C19	1.353 (8)
O7—C22	1.298 (6)	C18—H18A	0.9300
O8—C25	1.346 (7)	C19—C20	1.374 (9)
O8—C30	1.424 (8)	C20—C21	1.371 (7)
N1—C4	1.467 (7)	C20—H20A	0.9300
N2—C7	1.311 (6)	C21—H21A	0.9300
N2—N3	1.395 (5)	C23—C24	1.450 (7)
N3—C8	1.304 (5)	C23—H23A	0.9300
N4—C19	1.470 (8)	C24—C29	1.400 (7)
N5—C22	1.326 (6)	C24—C25	1.408 (7)
N5—N6	1.390 (5)	C25—C26	1.393 (8)
N6—C23	1.294 (6)	C26—C27	1.387 (8)
N7—C35	1.332 (6)	C26—H26A	0.9300
N7—C31	1.336 (6)	C27—C28	1.357 (9)
C1—C2	1.388 (6)	C27—H27A	0.9300
C1—C6	1.392 (6)	C28—C29	1.394 (7)
C1—C7	1.487 (7)	C28—H28A	0.9300
C2—C3	1.374 (6)	C29—H29A	0.9300
C2—H2B	0.9300	C30—H30A	0.9600
C3—C4	1.385 (7)	C30—H30B	0.9600
C3—H3A	0.9300	C30—H30C	0.9600
C4—C5	1.365 (7)	C31—C32	1.374 (6)
C5—C6	1.362 (7)	C31—H31A	0.9300
C5—H5B	0.9300	C32—C33	1.374 (7)
C6—H6A	0.9300	C32—H32A	0.9300
C8—C9	1.456 (7)	C33—C34	1.381 (8)
C8—H8A	0.9300	C33—C36	1.524 (7)
C9—C14	1.395 (7)	C34—C35	1.385 (7)
C9—C10	1.424 (7)	C34—H34A	0.9300
C10—C11	1.364 (8)	C35—H35A	0.9300
C11—C12	1.381 (9)	C36—H36A	0.9600
C11—H11A	0.9300	C36—H36B	0.9600
C12—C13	1.393 (9)	C36—H36C	0.9600
			
O7—Co1—O3	173.83 (15)	C9—C14—H14A	119.1
O7—Co1—N3	93.09 (15)	O4—C15—H15A	109.5
O3—Co1—N3	82.36 (15)	O4—C15—H15B	109.5
O7—Co1—N6	82.64 (17)	H15A—C15—H15B	109.5
O3—Co1—N6	93.15 (17)	O4—C15—H15C	109.5
N3—Co1—N6	90.00 (16)	H15A—C15—H15C	109.5
O7—Co1—N7	93.80 (15)	H15B—C15—H15C	109.5
O3—Co1—N7	90.72 (15)	C17—C16—C21	119.4 (5)
N3—Co1—N7	173.07 (18)	C17—C16—C22	119.7 (5)
N6—Co1—N7	90.18 (16)	C21—C16—C22	120.9 (5)
O7—Co1—Cl1	91.96 (12)	C18—C17—C16	120.2 (6)
O3—Co1—Cl1	92.19 (11)	C18—C17—H17A	119.9
N3—Co1—Cl1	89.84 (13)	C16—C17—H17A	119.9
N6—Co1—Cl1	174.59 (14)	C19—C18—C17	119.5 (6)
N7—Co1—Cl1	90.63 (12)	C19—C18—H18A	120.2
C7—O3—Co1	110.7 (3)	C17—C18—H18A	120.2
C10—O4—C15	117.6 (5)	C18—C19—C20	121.8 (6)
C22—O7—Co1	110.5 (3)	C18—C19—N4	119.1 (7)
C25—O8—C30	119.5 (6)	C20—C19—N4	119.2 (7)
O1—N1—O2	122.7 (5)	C21—C20—C19	119.1 (6)
O1—N1—C4	118.8 (5)	C21—C20—H20A	120.5
O2—N1—C4	118.4 (5)	C19—C20—H20A	120.5
C7—N2—N3	108.7 (4)	C20—C21—C16	120.1 (6)
C8—N3—N2	119.5 (4)	C20—C21—H21A	120.0
C8—N3—Co1	127.2 (4)	C16—C21—H21A	120.0
N2—N3—Co1	113.3 (3)	O7—C22—N5	124.6 (5)
O6—N4—O5	124.6 (7)	O7—C22—C16	118.5 (5)
O6—N4—C19	119.2 (8)	N5—C22—C16	116.9 (5)
O5—N4—C19	116.2 (8)	N6—C23—C24	131.7 (5)
C22—N5—N6	108.6 (4)	N6—C23—H23A	114.1
C23—N6—N5	119.3 (5)	C24—C23—H23A	114.1
C23—N6—Co1	127.2 (4)	C29—C24—C25	118.4 (6)
N5—N6—Co1	113.5 (3)	C29—C24—C23	125.6 (5)
C35—N7—C31	116.7 (4)	C25—C24—C23	116.0 (5)
C35—N7—Co1	122.3 (4)	O8—C25—C26	123.5 (6)
C31—N7—Co1	121.0 (3)	O8—C25—C24	116.7 (6)
C2—C1—C6	119.7 (5)	C26—C25—C24	119.8 (6)
C2—C1—C7	119.2 (4)	C27—C26—C25	120.0 (6)
C6—C1—C7	121.2 (4)	C27—C26—H26A	120.0
C3—C2—C1	120.4 (5)	C25—C26—H26A	120.0
C3—C2—H2B	119.8	C28—C27—C26	121.1 (7)
C1—C2—H2B	119.8	C28—C27—H27A	119.5
C2—C3—C4	117.9 (5)	C26—C27—H27A	119.5
C2—C3—H3A	121.1	C27—C28—C29	119.8 (6)
C4—C3—H3A	121.1	C27—C28—H28A	120.1
C5—C4—C3	122.8 (5)	C29—C28—H28A	120.1
C5—C4—N1	118.1 (5)	C28—C29—C24	120.9 (6)
C3—C4—N1	119.1 (5)	C28—C29—H29A	119.6
C6—C5—C4	118.9 (5)	C24—C29—H29A	119.6
C6—C5—H5B	120.6	O8—C30—H30A	109.5
C4—C5—H5B	120.6	O8—C30—H30B	109.5
C5—C6—C1	120.4 (5)	H30A—C30—H30B	109.5
C5—C6—H6A	119.8	O8—C30—H30C	109.5
C1—C6—H6A	119.8	H30A—C30—H30C	109.5
O3—C7—N2	124.8 (5)	H30B—C30—H30C	109.5
O3—C7—C1	118.0 (4)	N7—C31—C32	123.4 (5)
N2—C7—C1	117.2 (4)	N7—C31—H31A	118.3
N3—C8—C9	129.8 (5)	C32—C31—H31A	118.3
N3—C8—H8A	115.1	C33—C32—C31	120.1 (5)
C9—C8—H8A	115.1	C33—C32—H32A	119.9
C14—C9—C10	117.3 (5)	C31—C32—H32A	119.9
C14—C9—C8	126.8 (5)	C32—C33—C34	116.8 (5)
C10—C9—C8	115.9 (5)	C32—C33—C36	121.6 (6)
C11—C10—O4	123.9 (6)	C34—C33—C36	121.6 (6)
C11—C10—C9	121.0 (6)	C33—C34—C35	119.9 (6)
O4—C10—C9	115.0 (5)	C33—C34—H34A	120.1
C10—C11—C12	120.1 (6)	C35—C34—H34A	120.1
C10—C11—H11A	120.0	N7—C35—C34	123.0 (5)
C12—C11—H11A	120.0	N7—C35—H35A	118.5
C11—C12—C13	120.6 (6)	C34—C35—H35A	118.5
C11—C12—H12A	119.7	C33—C36—H36A	109.5
C13—C12—H12A	119.7	C33—C36—H36B	109.5
C14—C13—C12	119.2 (6)	H36A—C36—H36B	109.5
C14—C13—H13A	120.4	C33—C36—H36C	109.5
C12—C13—H13A	120.4	H36A—C36—H36C	109.5
C13—C14—C9	121.8 (6)	H36B—C36—H36C	109.5
C13—C14—H14A	119.1		
			
N3—Co1—O3—C7	2.5 (3)	C15—O4—C10—C9	175.4 (5)
N6—Co1—O3—C7	−87.1 (3)	C14—C9—C10—C11	−2.3 (8)
N7—Co1—O3—C7	−177.3 (3)	C8—C9—C10—C11	178.5 (5)
Cl1—Co1—O3—C7	92.1 (3)	C14—C9—C10—O4	−178.9 (5)
N3—Co1—O7—C22	−86.5 (3)	C8—C9—C10—O4	2.0 (7)
N6—Co1—O7—C22	3.1 (3)	O4—C10—C11—C12	178.1 (6)
N7—Co1—O7—C22	92.8 (3)	C9—C10—C11—C12	1.8 (9)
Cl1—Co1—O7—C22	−176.4 (3)	C10—C11—C12—C13	−0.6 (10)
C7—N2—N3—C8	−176.1 (4)	C11—C12—C13—C14	0.0 (11)
C7—N2—N3—Co1	2.8 (5)	C12—C13—C14—C9	−0.6 (11)
O7—Co1—N3—C8	−8.4 (4)	C10—C9—C14—C13	1.7 (9)
O3—Co1—N3—C8	175.8 (4)	C8—C9—C14—C13	−179.3 (6)
N6—Co1—N3—C8	−91.0 (4)	C21—C16—C17—C18	−0.1 (9)
Cl1—Co1—N3—C8	83.6 (4)	C22—C16—C17—C18	−178.0 (5)
O7—Co1—N3—N2	172.8 (3)	C16—C17—C18—C19	0.6 (9)
O3—Co1—N3—N2	−3.0 (3)	C17—C18—C19—C20	−0.3 (10)
Cl1—Co1—N3—N2	−95.2 (3)	C17—C18—C19—N4	−179.2 (6)
C22—N5—N6—C23	−179.7 (4)	O6—N4—C19—C18	10.4 (10)
C22—N5—N6—Co1	1.0 (4)	O5—N4—C19—C18	−170.5 (7)
O7—Co1—N6—C23	178.4 (4)	O6—N4—C19—C20	−168.5 (7)
O3—Co1—N6—C23	−6.1 (4)	O5—N4—C19—C20	10.6 (10)
N3—Co1—N6—C23	−88.5 (4)	C18—C19—C20—C21	−0.5 (10)
N7—Co1—N6—C23	84.6 (4)	N4—C19—C20—C21	178.4 (5)
O7—Co1—N6—N5	−2.3 (3)	C19—C20—C21—C16	1.0 (9)
O3—Co1—N6—N5	173.1 (3)	C17—C16—C21—C20	−0.7 (8)
N3—Co1—N6—N5	90.8 (3)	C22—C16—C21—C20	177.2 (5)
N7—Co1—N6—N5	−96.1 (3)	Co1—O7—C22—N5	−3.8 (5)
O7—Co1—N7—C35	0.5 (4)	Co1—O7—C22—C16	174.8 (3)
O3—Co1—N7—C35	176.3 (4)	N6—N5—C22—O7	1.9 (6)
N6—Co1—N7—C35	83.1 (4)	N6—N5—C22—C16	−176.8 (4)
Cl1—Co1—N7—C35	−91.5 (4)	C17—C16—C22—O7	−18.2 (7)
O7—Co1—N7—C31	−179.1 (4)	C21—C16—C22—O7	163.9 (5)
O3—Co1—N7—C31	−3.3 (4)	C17—C16—C22—N5	160.5 (5)
N6—Co1—N7—C31	−96.5 (4)	C21—C16—C22—N5	−17.4 (7)
Cl1—Co1—N7—C31	88.9 (4)	N5—N6—C23—C24	−3.0 (7)
C6—C1—C2—C3	−0.5 (8)	Co1—N6—C23—C24	176.2 (4)
C7—C1—C2—C3	177.6 (5)	N6—C23—C24—C29	1.5 (8)
C1—C2—C3—C4	0.0 (8)	N6—C23—C24—C25	−178.8 (5)
C2—C3—C4—C5	1.5 (9)	C30—O8—C25—C26	6.5 (9)
C2—C3—C4—N1	−178.0 (5)	C30—O8—C25—C24	−174.6 (6)
O1—N1—C4—C5	6.1 (9)	C29—C24—C25—O8	178.4 (5)
O2—N1—C4—C5	−176.1 (6)	C23—C24—C25—O8	−1.3 (7)
O1—N1—C4—C3	−174.3 (6)	C29—C24—C25—C26	−2.6 (7)
O2—N1—C4—C3	3.5 (9)	C23—C24—C25—C26	177.7 (5)
C3—C4—C5—C6	−2.5 (9)	O8—C25—C26—C27	−179.5 (5)
N1—C4—C5—C6	177.1 (5)	C24—C25—C26—C27	1.6 (8)
C4—C5—C6—C1	1.9 (8)	C25—C26—C27—C28	0.5 (9)
C2—C1—C6—C5	−0.4 (8)	C26—C27—C28—C29	−1.5 (9)
C7—C1—C6—C5	−178.5 (5)	C27—C28—C29—C24	0.5 (8)
Co1—O3—C7—N2	−1.8 (6)	C25—C24—C29—C28	1.6 (7)
Co1—O3—C7—C1	179.2 (3)	C23—C24—C29—C28	−178.7 (5)
N3—N2—C7—O3	−0.7 (7)	C35—N7—C31—C32	−2.6 (8)
N3—N2—C7—C1	178.4 (4)	Co1—N7—C31—C32	177.0 (4)
C2—C1—C7—O3	−2.3 (7)	N7—C31—C32—C33	2.1 (8)
C6—C1—C7—O3	175.7 (5)	C31—C32—C33—C34	−0.4 (8)
C2—C1—C7—N2	178.6 (5)	C31—C32—C33—C36	−178.6 (5)
C6—C1—C7—N2	−3.4 (7)	C32—C33—C34—C35	−0.7 (8)
N2—N3—C8—C9	−1.9 (8)	C36—C33—C34—C35	177.6 (5)
Co1—N3—C8—C9	179.3 (4)	C31—N7—C35—C34	1.5 (8)
N3—C8—C9—C14	4.1 (10)	Co1—N7—C35—C34	−178.1 (4)
N3—C8—C9—C10	−176.9 (5)	C33—C34—C35—N7	0.1 (9)
C15—O4—C10—C11	−1.0 (8)		
